# Determination of the electronic portal imaging device pixel‐sensitivity‐map for quality assurance applications. Part 2: Photon beam dependence

**DOI:** 10.1002/acm2.13602

**Published:** 2022-04-15

**Authors:** Michael Paul Barnes, Baozhou Sun, Brad Michael Oborn, Bishnu Lamichhane, Stuart Szwec, Matthew Schmidt, Bin Cai, Frederick Menk, Peter Greer

**Affiliations:** ^1^ Department of Radiation Oncology Calvary Mater Hospital Newcastle Newcastle NSW Australia; ^2^ School of Mathematical and Physical Sciences University of Newcastle Newcastle NSW Australia; ^3^ Department of Radiation Oncology Washington University in St Louis St Louis Missouri USA; ^4^ Centre for Medical Radiation Physics University of Wollongong Wollongong NSW Australia; ^5^ Illawarra Cancer Care Centre Wollongong Hospital Wollongong NSW Australia; ^6^ School of Medicine and Public Health University of Newcastle Newcastle NSW Australia

**Keywords:** EPID dosimetry, pixel‐sensitivity‐map, quality assurance

## Abstract

**Purpose:**

The EPID PSM is a useful EPID calibration method for QA applications. The dependence of the EPID PSM on the photon beam used to acquire it has been investigated in this study for the four available PSM methods. The aim is to inform upon the viability of applying a single PSM for all available photon beams to simplify PSM implementation and maintenance.

**Methods:**

Four methods of PSM determination were each measured once in a single session on a single TrueBeam ® STx linac using 6 MV, 10 MV, 6 MV Flattening‐Filter‐Free (FFF), and 10 MV FFF photon beams. The resultant PSM was assessed for both intra‐ and inter‐method beam dependence via comparison between PSM of the same method compared to the 6 MV PSM and via comparison between PSM of the same beam with the corresponding Monte Carlo PSM. Comparisons were performed via 2D percentage deviation plots with associated histograms, 1D crossplane profiles, and via mean, median, and standard deviation percentage deviation statistics. Generated beam‐response was compared qualitatively via 1D crossplane profile comparison and quantitatively via symmetry assessment with comparison to the IC profiler device.

**Results:**

The Varian method provided the most consistent PSM with varying photon beam, with median percent deviation from the 6 MV PSM within 0.14% for all other beams. Qualitatively, each method provided similar beam‐response profiles. The measured beam‐response symmetry agreed to within 0.2% between the Calvary Mater Newcastle (CMN) method and IC profiler, but agreement reduced to within 0.9% and 2.2% for the Varian and WashU methods. PSM percent deviation with Monte Carlo PSM was within 0.75% for all methods and beams.

**Conclusion:**

Results suggest that the PSM may be independent of photon beam to clinically relevant levels. The Varian method of PSM determination introduces the least beam dependence into the measured PSM.

## 1 INTRODUCTION

The amorphous silicon (aSi) electronic portal imaging device (EPID) was designed for patient positioning applications. For such applications, the image non‐uniformities caused by the patient anatomy in the beam are of interest. As such, correcting out the non‐uniformities introduced by the beam and those introduced by the EPID panel itself are required. This is standardly achieved via the flood‐field calibration procedure. However, correcting out the non‐uniformities introduced by the beam is problematic for many linac and patient quality assurance (QA) applications because the beam non‐uniformities (i.e., profile shape) are the information required for investigation. As such, the flood‐field correction represents a major current limiting factor to the use of EPID for dosimetry and QA applications.

Because of the problems associated with the EPID flood‐field calibration for QA applications a number of authors have attempted to develop alternate EPID calibration procedures where the non‐uniformity of the imager response is corrected without disturbing the non‐uniformity of the incident beam.^1^, ^2^, ^3^, ^4^ Such a calibration procedure was first attempted by *Greer* ^1^ who named this new calibration procedure the pixel‐sensitivity‐map (PSM). The PSM is analogous to the array‐type calibrations used in commercial 2D‐array detectors to correct for response differences between individual detectors.

Three studies have been published which examined the utilization of PSM‐corrected EPID images for linac QA purposes.^5^, ^6^, ^7^ The study of Yaddanapudi et al.^5^ utilized PSM‐corrected EPID images for linac acceptance testing purposes. The study used changes in the flatness of the beam profile as measured using PSM‐corrected EPID imaging as a measure of beam energy. This study also suggested that the method could be used for beam symmetry evaluation, although no assessment of symmetry was presented. The study of Cai et al.^6^ also looked at QA applications of PSM‐corrected EPID images. The focus of that study was to demonstrate that PSM‐corrected EPID imaging could provide consistent profiles for matched linacs. Both the *Yaddanapudi and Cai* studies were based upon an adaptation of the *Boriano* method^2^ of determining the PSM. The study of Barnes et al.^7^ used PSM‐corrected EPID imaging as an absolute measure of wide field beam symmetry as a means of photon beam angle steering. The PSM used in the *Barnes* publication is based upon a simplified version of the *Greer* ^1^ method of PSM correction.

Similar to the linac QA applications, PSM calibration has demonstrated utility in EPID‐based patient‐specific QA applications.^8^, ^9^, ^10^ Unlike flood‐field corrected EPID images, correcting for the PSM would allow for first principles‐type dose‐to‐water conversion of EPID images that would allow for more direct comparison between patient QA EPID images and the treatment plans. The image would first be corrected by the PSM to remove the EPID introduced image non‐uniformities while preserving the profile shape, which can subsequently be converted to dose‐to‐water.

The question as to whether the PSM is dependent on the photon beam used to acquire it has not been definitively answered. In the study of Cai et al.,^6^ PSMs were generated from multiple‐flattened photon beams and electron beams. The raw images were then PSM‐corrected and analyzed. However, the PSMs generated from the different photon beams were not directly compared to inform on whether there was a beam dependence to the PSM or whether the PSM generated from one photon beam could be used to correct images from any beam. In the study of Ahmad et al., ^11^ a new method of PSM determination was presented. Comparison of PSM generated using different beams suggested that the PSM was independent of the beam.

If the PSM were independent of photon beam then this would make implementation simpler as only a single PSM generated from a single photon beam would need to be determined and maintained, but could be applied to all applicable QA measurements regardless of photon beam. It is the purpose of this Part 2 study to investigate the beam dependence of the PSM and of four available methods for determining it. Analysis using four different photon beams with each of the PSM methods allows for both the intra‐ and inter‐method beam dependence to be assessed.

## 2 METHODS

The PSM methods investigated include an improved Greer method henceforth known as the CMN method, a modified Boriano method henceforth known as the WashU method and a method developed by Varian (Varian Medical System, Palo Alto, CA, USA) henceforth known as the Varian method. A fourth method is also included based on data obtained from Monte Carlo simulations (the Monte Carlo method). Other methods of determining the PSM have been published in the literature,^3^, ^4^, ^11^ but are not included in this study. Details on the methodology of the four OSM methods investigated were presented in Part 1 of this study.^12^ Each empirical method was applied for multiple MV photon beams: 6 MV and 10 MV and 6 and 10 MV FFF. In the case of the Monte Carlo method, the beam‐response simulations were repeated for each beam. It is reminded that the Varian method is not currently commercially available.

### 2.1 Materials

The measurements in this study were performed on a single Varian TrueBeam ® STx linac with aS1200 EPID. The aS1200 panel utilizes a 43 × 43 cm^2^ panel with 40 × 40 cm^2^ active area in an 1190 × 1190 pixel array used for dosimetry mode resulting in resolution of 0.34 mm at isocenter. The aS1200 EPID also has a backscatter plate to remove backscatter from the EPID support arm, which would otherwise introduce an additional source of non‐uniformity to be corrected by the PSM. Results’ comparison was performed using a custom Matlab script (Mathworks Inc., Natick, MA, USA).

### 2.2 Measurement methods

#### 2.2.1 Data collection

Data were collected for all four PSM methods in a single measurement session. Each method was performed once on each of the available photon beams (6 and 10 MV and 6 and 10 MV FFF) so that an assessment of PSM dependence on photon beam could be made. Use of a single measurement is justified by the high short‐term repeatability observed for all methods in Part 1 of this study.^12^ The CMN method and the Monte Carlo method directly result in the determination of the beam‐response, while the Varian and WashU methods result in the PSM being determined. In the measurement session, wide‐field EPID images were also taken for each beam. The applied flood field for each image was removed to leave the raw EPID image. For the Monte Carlo and CMN methods, the measured beam‐response was removed from the raw image to provide the PSM, while for the WashU and Varian methods the determined PSM was removed from the raw images to provide the measured beam‐response. In this way, beam‐responses and PSMs were determined for each method for comparison.

#### 2.2.2 Beam dependence

##### Intra‐method beam dependence

If the actual PSM is beam independent, it is possible that the methods of determining it introduce a beam dependence into the results. To inform on these questions, the measured PSM per beam were plotted as 2D percentage deviation maps with corresponding histograms relative to the 6 MV PSM. This was repeated for each PSM method. Additionally, 1D crossplane profiles for each beam were all plotted together per method, together with percentage deviation from the 6MV PSM for each method, so that results for all methods could be compared in the same plot.

##### Inter‐method beam dependence

In Part 1 of this study,^12^ the PSM results for the three empirical methods were compared against results from the Monte Carlo method for the 6 MV beam. In Part 2 of this study, this analysis has been extended to the other available photon beams. For brevity, the PSM 2D percentage deviation plots and histograms along with the 1D profile comparisons have been included as supplementary material and only the percent deviation statistics are presented in the main body of the text.

The beam‐response generated by each PSM method and for each energy was assessed both qualitatively and quantitatively. Qualitatively, 1D beam‐response crossplane profiles were overlaid and visually contrasted. Quantitatively, Sun Nuclear IC Profiler (Sun Nuclear Corporation, Melbourne, FL) measurements of beam symmetry using the Point Ratio (IEC 976) metric were performed for each beam. Symmetry of the beam‐response in both inplane and crossplane directions was calculated using the IEC 976 definition of symmetry^13^ for each PSM method, and compared against the IC Profiler measured symmetry to provide a clinically significant assessment of each PSM method. Due to the overresponse of the aSi panel to the low energy spectrum of the beam, which results in exaggerated beam horns, the flatness metric could not be compared to an external dose‐to‐water method.

##### Dose rate dependency

The aim of this study is to inform on whether the PSM generated with a single photon beam can be used for other available photon beams. For this to be feasible the PSM would need to be independent of a number of photon beam parameters that differ between beams. Such parameters include, but may not necessarily be limited to, beam spectrum and dose rate. As an initial investigation into the dose rate dependency a simple experiment was performed whereby in a single measurement session the PSM was generated using the CMN method for the 10 MV FFF beam with nominal dose rate set to both 400 MU/min, representing the minimum available nominal dose rate and 2400 MU/min representing the maximum available nominal dose rate. This beam was chosen as the one with the greatest variation in available nominal dose rate. If there were to be a dependency on dose rate than this may manifest in either the beam‐response or PSM. As such, both were investigated in this experiment. Both the beam‐response and PSM were generated for each dose rate using the CMN method and compared via 2D Percent deviation maps and percentage deviation histograms.

## 3 RESULTS

**FIGURE 1:**
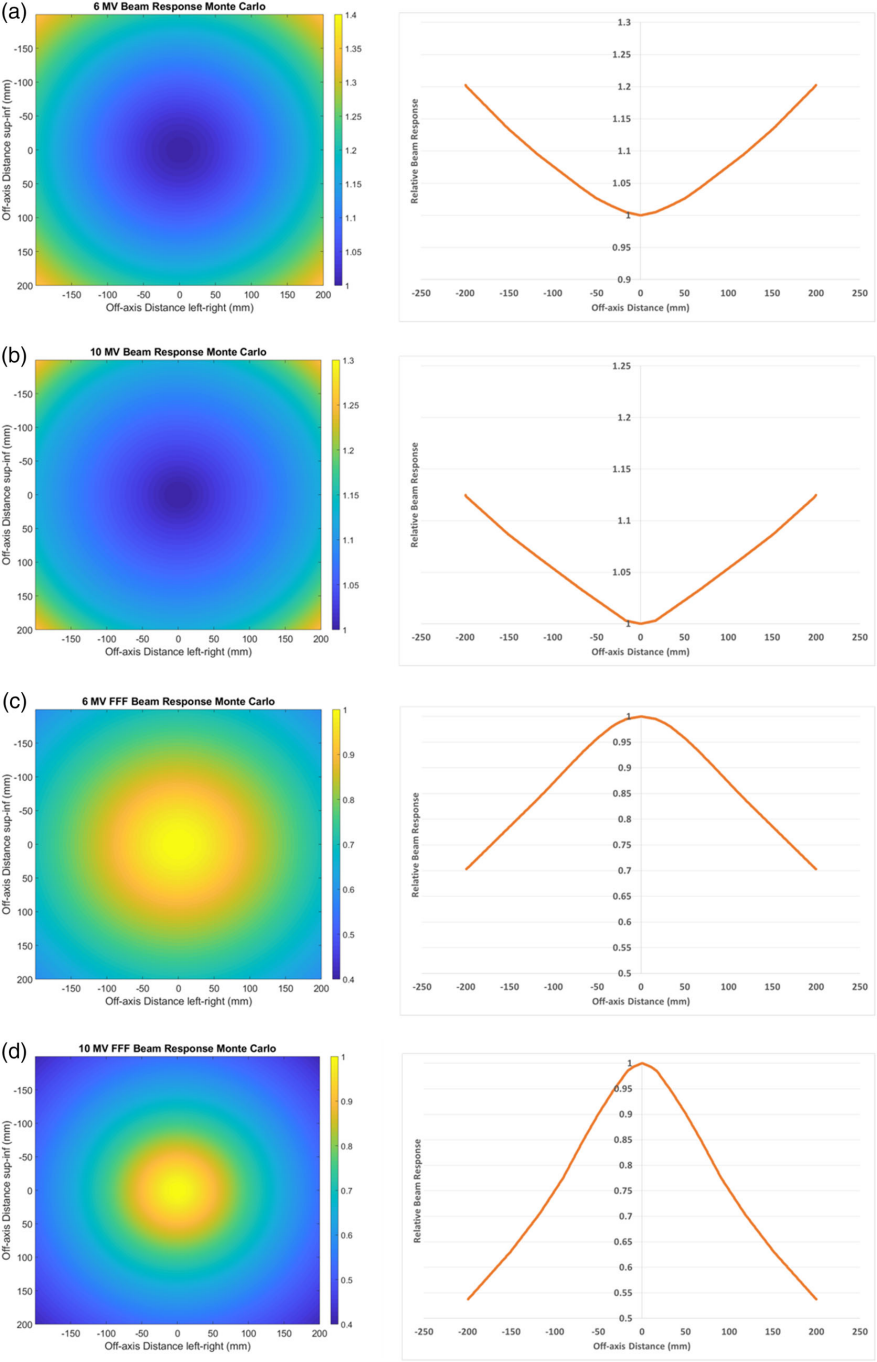
Beam‐response in both two‐dimensional (2D) (left) and 1D central axis crossplane profile right as determined with the Monte Carlo method. (a) 6 MV, (b) 10 MV, (c) 6 MV FFF, and (d) 10 MV FFF. Data are normalized to the central axis

Figure 1 provides examples of the beam‐response with different photon energies for the Monte Carlo method as a demonstration of the difference of the beam‐response with photon beam.

### 3.1 Intra‐method pixel‐sensitivity‐map variability with photon beam

**FIGURE 2:**
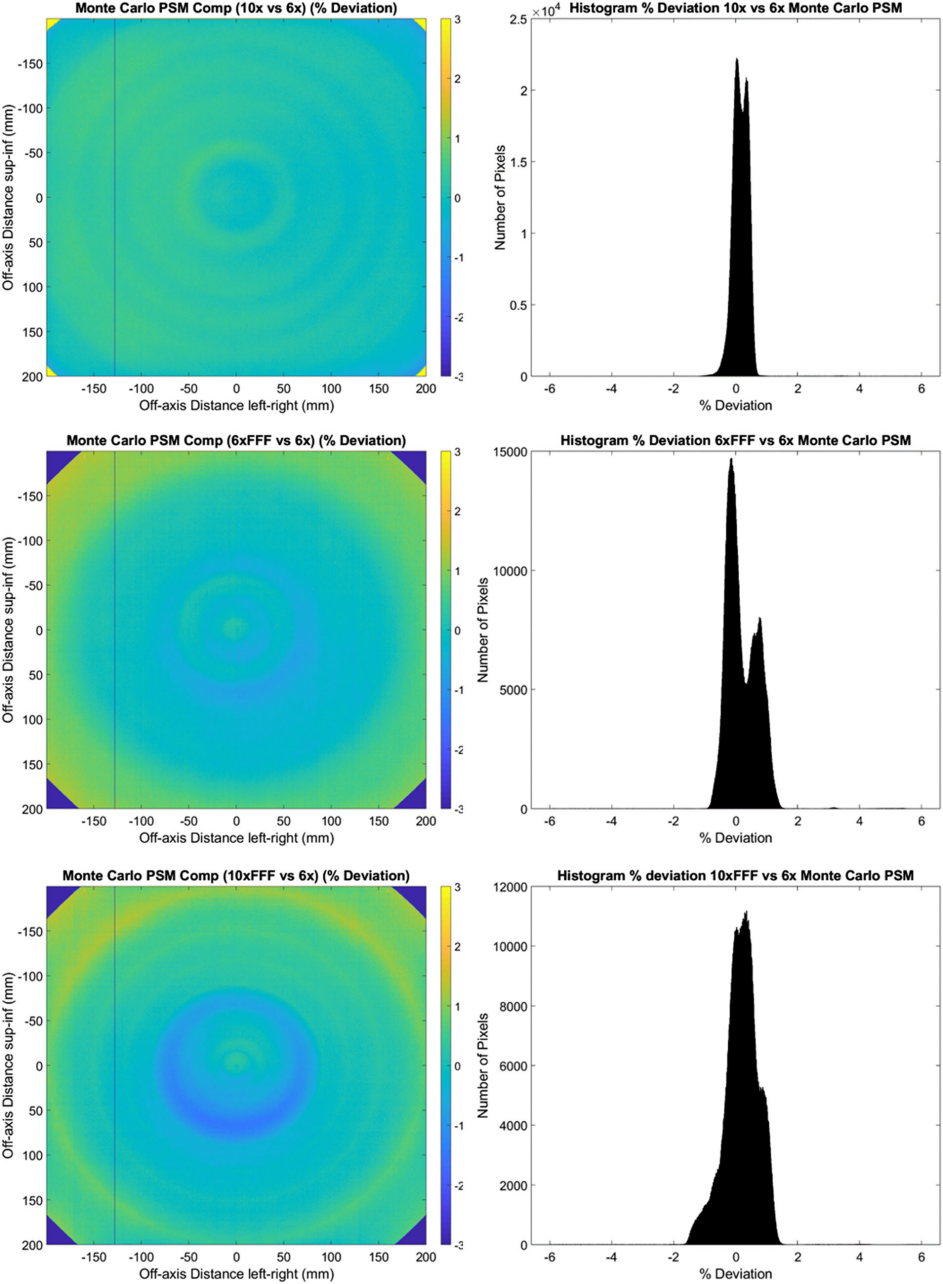
Monte Carlo 2D% deviation in pixel‐sensitivity‐map (PSM) between each beam compared to the 6 MV beam result. (10 MV top, 6 MV FFF middle, and 10 MV FFF bottom). % Deviation maps (left) and corresponding histograms (right)

Figure 2 shows the percent deviation between the PSM measured with each beam for the Monte Carlo method. The histograms demonstrate that for all beams the majority of pixels agree within ± 2% with the most consistent agreement observed with the 10 MV beam, where the majority of pixels are within ± 1%. The histograms are centered about 0% deviation. These findings are confirmed with the statistics presented in Table 1 with the worst results measured at −0.33%, 0.21%, and 4.69% for mean, median, and standard deviation, respectively. The 2D percentage deviation plots presented on the left‐hand side of Figure 2 show a ring effect, which may be attributable to error introduced by the radial averaging applied to the Monte Carlo beam‐response data. The effect is most pronounced in comparing the 10 MV FFF and 6 MV beam in the bottom plot of Figure 2. The 2D percentage deviation plots also show the best agreement in the corners of the image, where the primary collimator has effect, between the 6 and 10 MV beams. This could potentially indicate a difference in how the Monte Carlo is being modeled in these regions between flattened and FFF beams.

**TABLE 1:** Intra‐method pixel‐sensitivity‐map (PSM) percent deviation mean, median and standard deviation compared with the 6 MV result from the corresponding method

		**%Deviation**		
**Method**	**Photon beam (MV)**	**Mean**	**Median**	**SD**
Monte Carlo	6 FFF	−0.33	0.07	4.69
	10	0.18	0.16	0.56
	10 FFF	−0.23	0.21	3.94
CMN	6 FFF	−0.75	−0.17	4.70
	10	0.40	0.29	0.81
	10 FFF	−0.27	0.17	3.87
Varian	6 FFF	−0.05	−0.04	0.11
	10	0.15	0.14	0.16
	10 FFF	0.04	0.03	0.15
WashU	6 FFF	−1.36	−1.10	1.17
	10	−0.05	−0.04	0.55
	10 FFF	−1.72	−1.35	1.46

**FIGURE 3:**
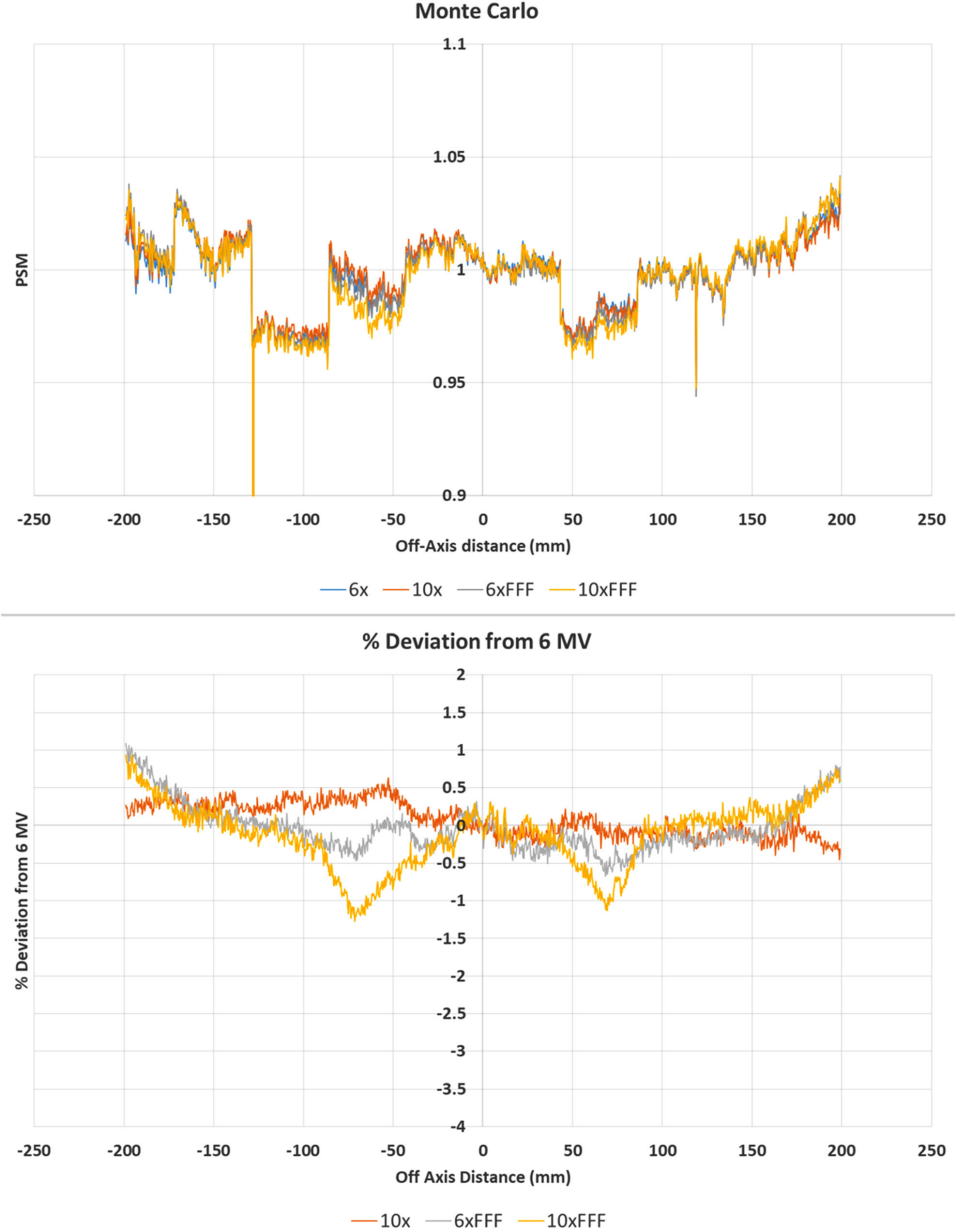
Central axis crossplane profiles for the pixel‐sensitivity‐map (PSM) determined using the Monte Carlo method for each available photon beam. Crossplane PSM profile (top) and crossplane PSM profile percent deviation from 6 MV (bottom)

**FIGURE 4:**
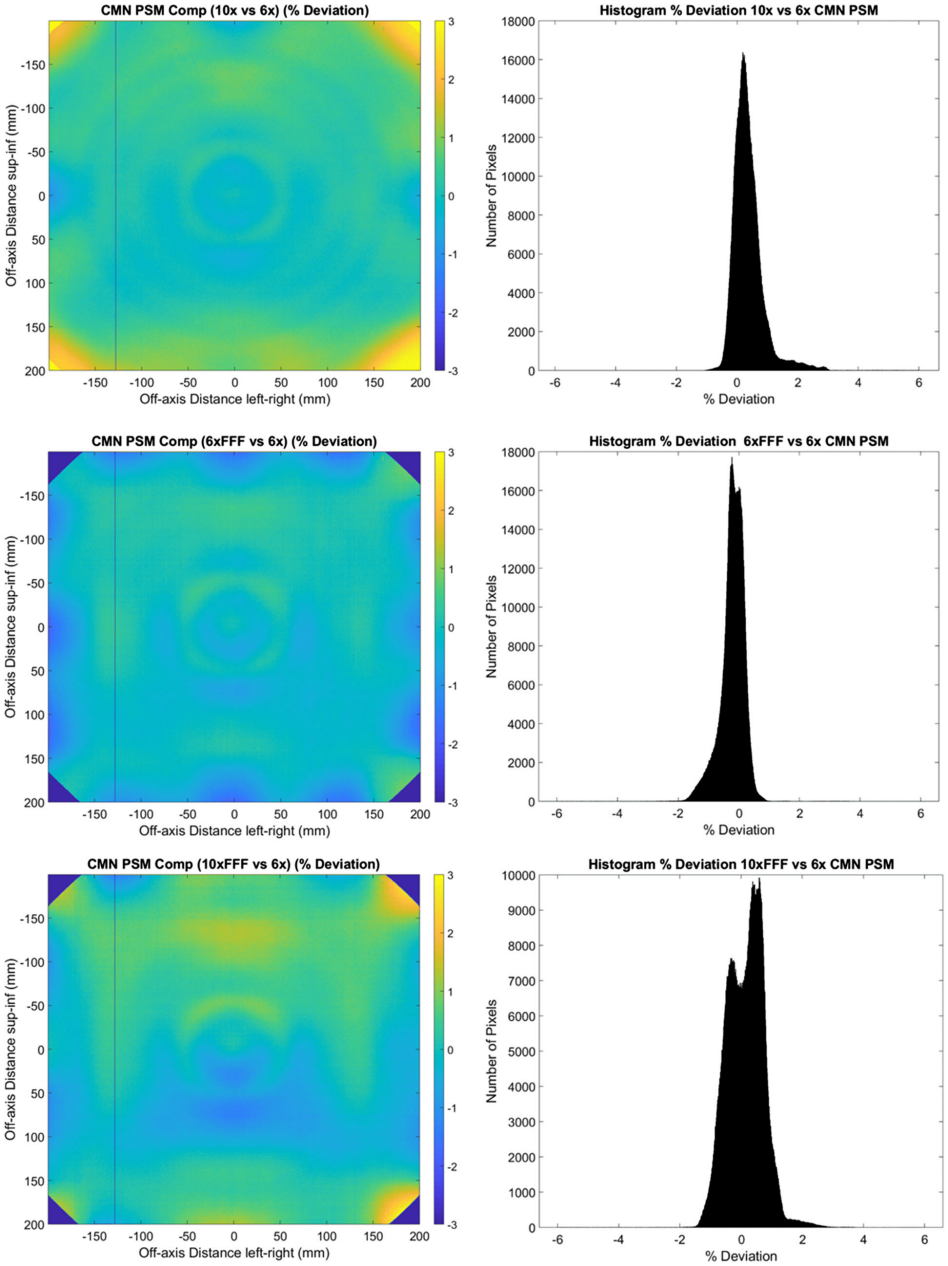
CMN 2D % deviation in pixel‐sensitivity‐map (PSM) between each beam compared to the 6 MV beam result. (10 MV top, 6 MV FFF middle, and 10 MV FFF bottom). % Deviation maps (left) and corresponding histograms (right)

**FIGURE 5:**
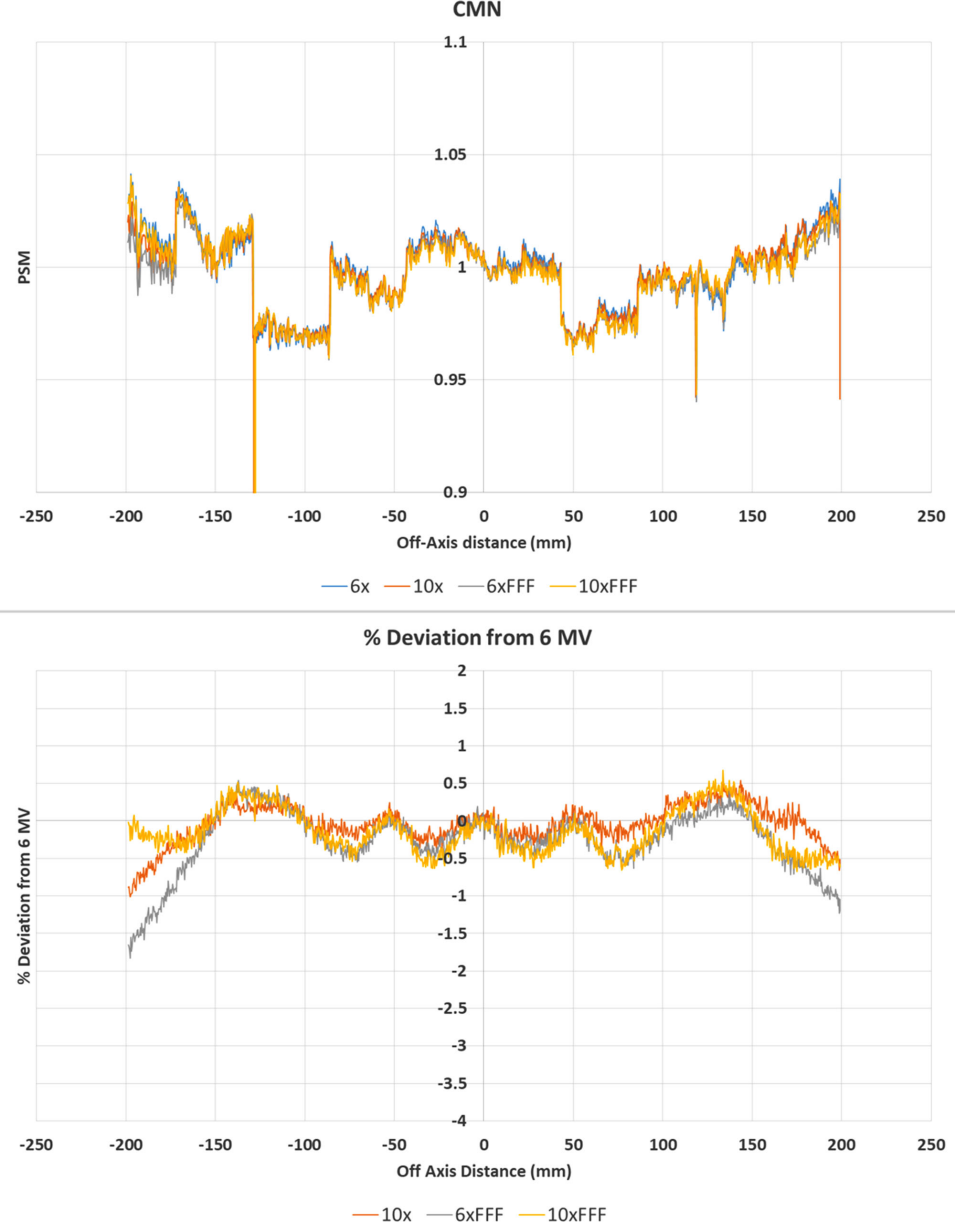
Central axis crossplane profiles for the pixel‐sensitivity‐map (PSM) determined using the CMN method from each available photon beam. Crossplane PSM profile (top) and crossplane PSM profile percent deviation from 6 MV (bottom)

**FIGURE 6:**
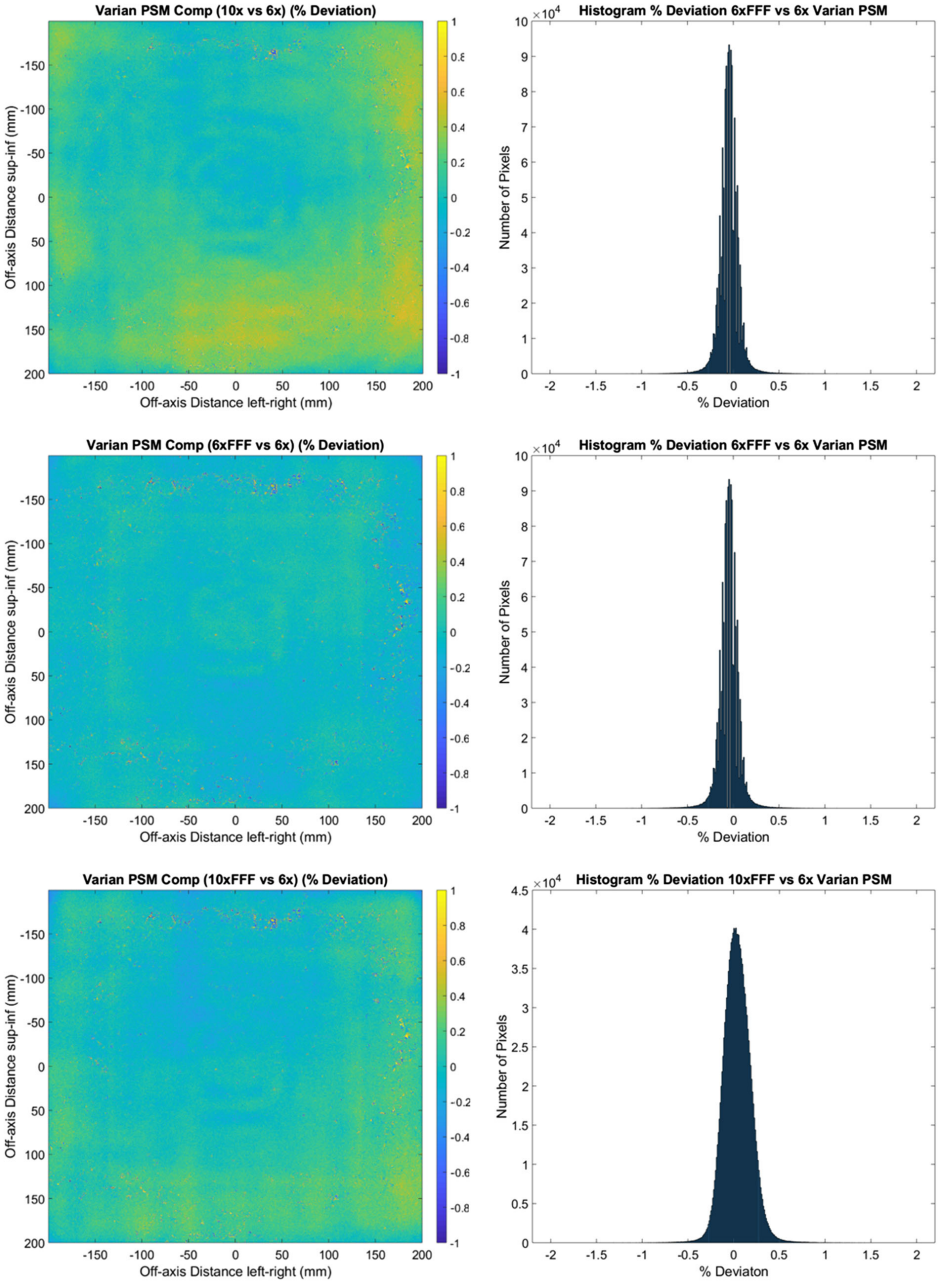
Varian two‐dimensional (2D) % deviation in pixel‐sensitivity‐map (PSM) between each beam compared to the 6 MV beam result. (10 MV top, 6 MV FFF middle, and 10 MV FFF bottom). % Deviation maps (left) and corresponding histograms (right). Note the reduced scales on the plots compared to those in corresponding plots (Figures 2, 4, and 8) from different methods

**FIGURE 7:**
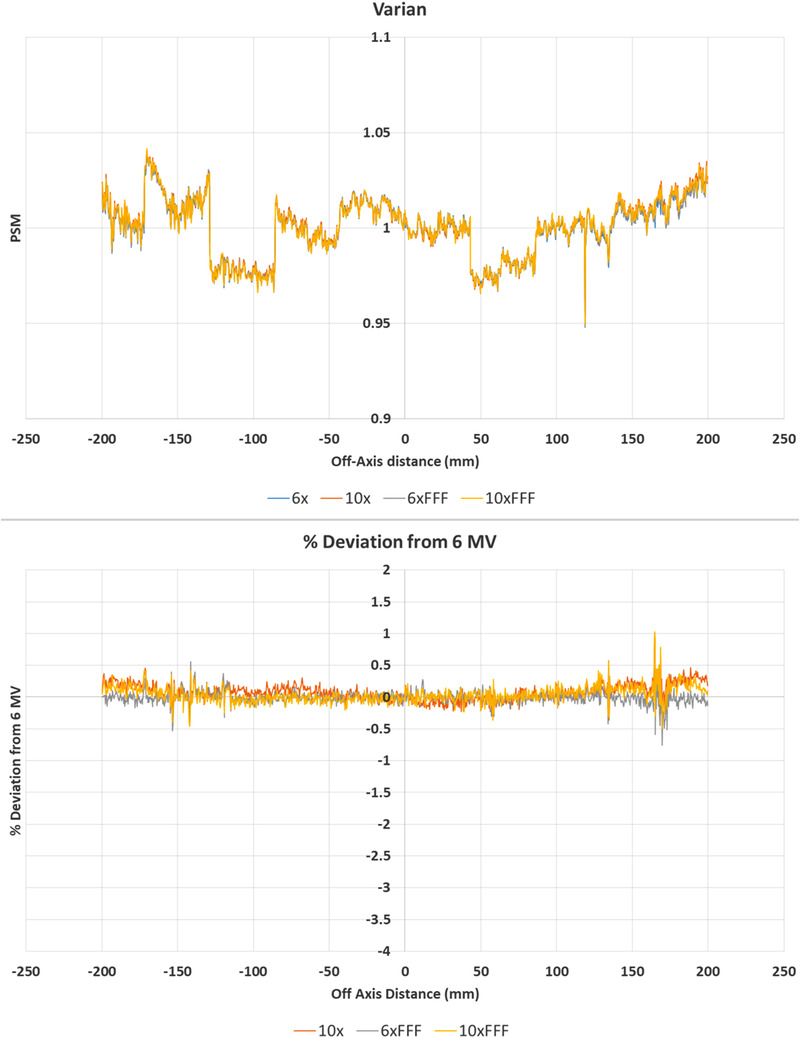
Central axis crossplane profiles for the pixel‐sensitivity‐map (PSM) determined using the Varian method from each available photon beam. Crossplane PSM profile (top) and crossplane PSM profile percent deviation from 6 MV (bottom)

**FIGURE 8:**
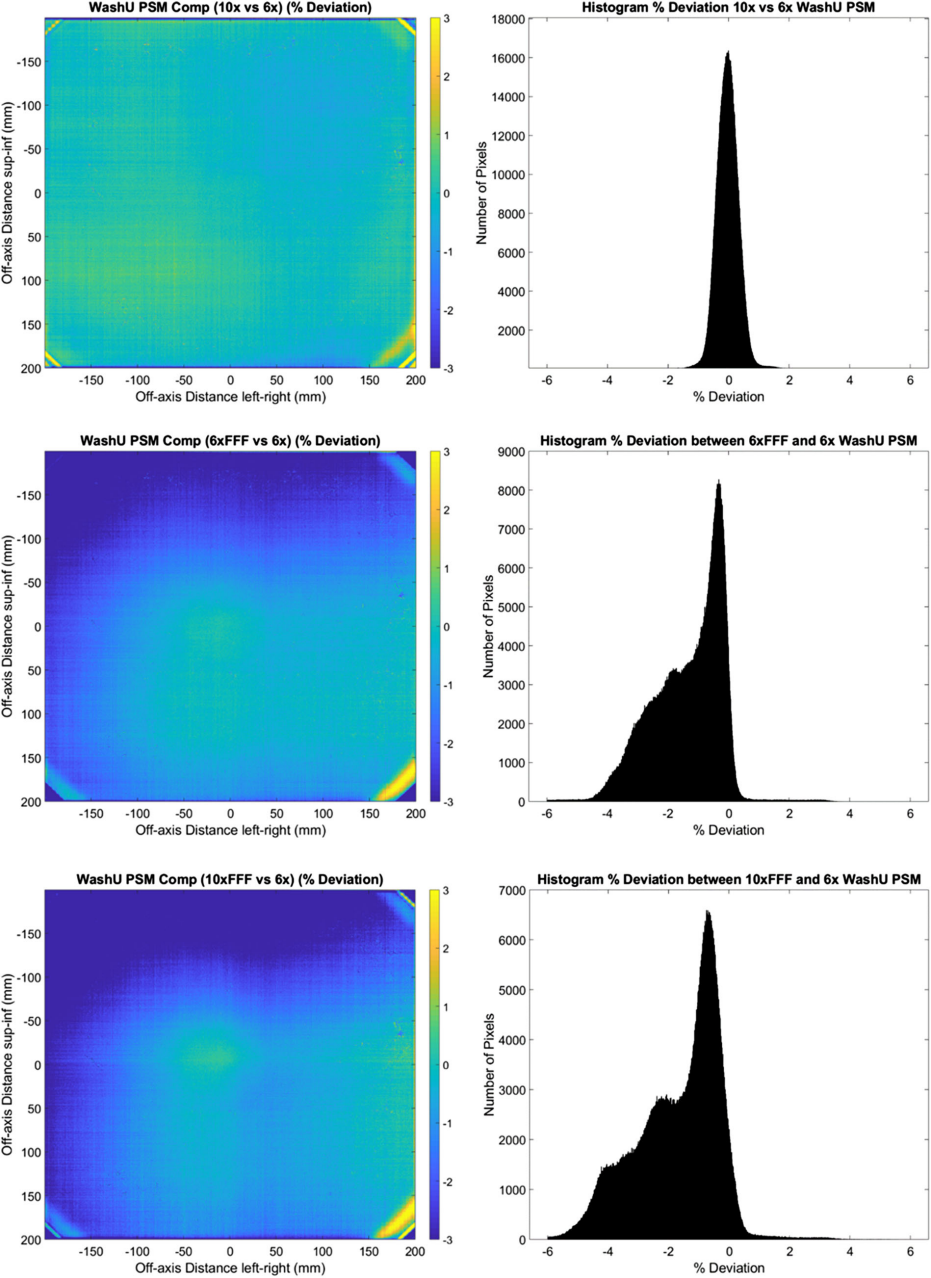
WashU 2D % deviation in pixel‐sensitivity‐map (PSM) between each beam compared to the 6 MV beam result. (10 MV top, 6 MV FFF middle, and 10 MV FFF bottom). % Deviation maps (left) and corresponding histograms (right)

Figure 3 provides a means of being able to compare the consistency of PSM across beams for the Monte Carlo method with all beam results on the same plot. Results are similarly presented for the other PSM methods in Figure 4, 5, 6, 7, 8, 9. The top plot of Figure 3 shows qualitatively good agreement between the central axis crossplane PSM measured by the Monte Carlo method for all four beams. Inferior agreement for the 10 MV FFF PSM symmetrically between 50 and 100 mm off axis is observed, where the disagreement with the 6 MV beam ‘spikes’ down to approximately −1.3% before improving again further off axis to be within 0.5% again. These ‘spikes’ of disagreement coincide with the rings effects observed in Figure 2, which suggest inaccuracy in the Monte Carlo 10 MV FFF beam‐response. Outside ± 150 mm off‐axis agreement declines up to approximately 1% at 200 mm off‐axis. However, this section of the image is of least utility for QA applications.

**FIGURE 9:**
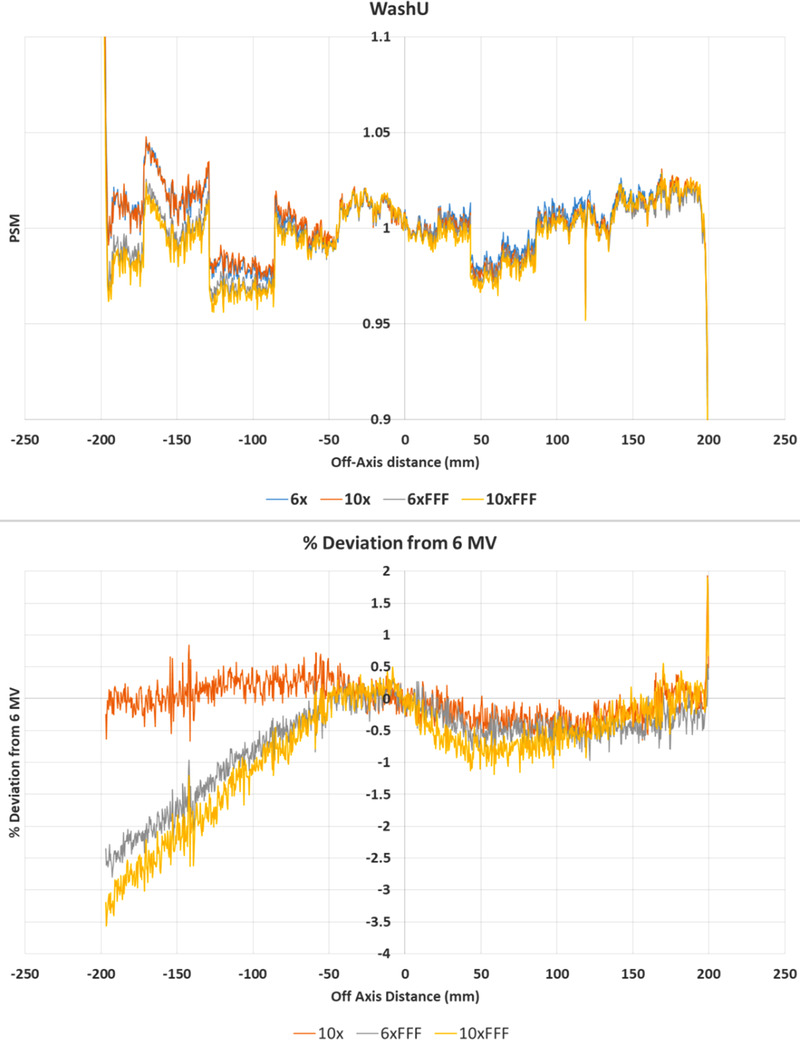
Central axis crossplane profiles for the pixel‐sensitivity‐map (PSM) determined using the WashU method from each available photon beam. Crossplane PSM profile (top) and crossplane PSM profile percent deviation from 6 MV (bottom)

Figure 4 shows the agreement between the PSM measured with each beam for the CMN method. The histograms show agreement for the majority of pixels for each beam within ± 2% compared to the 6 MV PSM. The statistics of Table 1 indicate worse agreement than the Monte Carlo method with percent deviation mean, median, and standard deviation measured at −0.75, −0.17, and 4.70%, respectively. The 2D percentage deviation plots on the left‐hand side of Figure 4 show a similar ring effect, but reduced in magnitude, to the Monte Carlo results of Figure 2. The CMN method does not utilize radial averaging and hence this cannot be the cause of this ring effect. The qualitative similarity of the ring effect in Figures 2 and 4 cast doubt on radial averaging being the cause of the ring effect in the Monte Carlo results. Similar results are observed in the primary collimator regions for the CMN method as are observed in the Monte Carlo results.

The top plot of Figure 5 shows qualitatively good agreement between the central axis crossplane PSM measured by the CMN method for all four beams. Within ± 150 mm, a sinusoidal‐type pattern of agreement is observed with best agreement observed every 50 mm. It is at these 50 mm off‐axis points that the PSM is directly measured. As such, such good agreement is to be expected and suggests that the underlying PSM is beam independent. In between the measurement points percent deviation drops to within 0.5%. While still considered to be good agreement this indicates that the fit applied to the directly measured points could potentially be improved. Outside ± 150 mm the agreement with the 6 MV beam declines for all beams with nearly 2% disagreement observed in the case of the 6 MV FFF beam. This region is outside the direct measurement data points and hence the fit is an extrapolation. An extra directly measured data point outside ± 150 mm could improve this, but runs into edge effects as the 5 × 5 cm field used to directly measure the PSM may no longer fully fit onto the EPID panel. The extremities of the panel are of least importance for QA applications, many of which could be performed on a 30 × 30 cm^2^ field within the directly measured PSM data points.

Figure 6 and Table 1 show that the Varian method provides the most consistent PSM between beams, with percent deviation for all energies within ± 0.5% for the majority of pixels. Note that the scales on both the 2D percentage deviation and histogram plots have been reduced compared to results from the other methods for visualization purposes. The superior agreement demonstrated in the histograms for the Varian method is reinforced by the values in Table 1, with worse‐case percent deviation measured at 0.15, 0.14, and 0.16 for mean, median, and standard deviation, respectively. These results provide evidence that the underlying PSM is beam independent.

Figure 7 reinforces the low beam dependence of the PSM generated using the Varian method. The exception to high agreement between beams is between 150 and 200 mm off‐axis, where spikes are observed of disagreement up to 1%. This is also observed in the 2D percentage deviation plots of Figure 6, where a speckled pattern is observed in the corresponding areas. The cause of this region of relatively higher disagreement is unknown, but may be due to pixel instability, which is within the uncertainty of other methods and hence not significant.

Figures 8 and 9 indicate that the WashU method is the most beam dependent of all the methods examined. This is particularly the case on the negative off‐axis direction, where systematic offsets are observed. Of note is that the flattened beam PSMs appear to agree with each other and the FFF PSMs appear to agree with one another, but that the FFF and flattened beams do not agree. This is evident in the histogram results where for the 10 MV results the majority of pixels are within ± 1% of the 6 MV results, but in comparing the FFF beam results to the 6 MV beam agreement is only within approximately −5 to +1%. The relatively high beam dependence of the WashU method is also demonstrated in the statistics, with worse cases measured at −1.72, −1.35, and 1.44% for mean, median, and standard deviation, respectively.

### 3.2 Inter‐method beam dependence

#### 3.2.1 Beam‐response beam dependence

**FIGURE 10:**
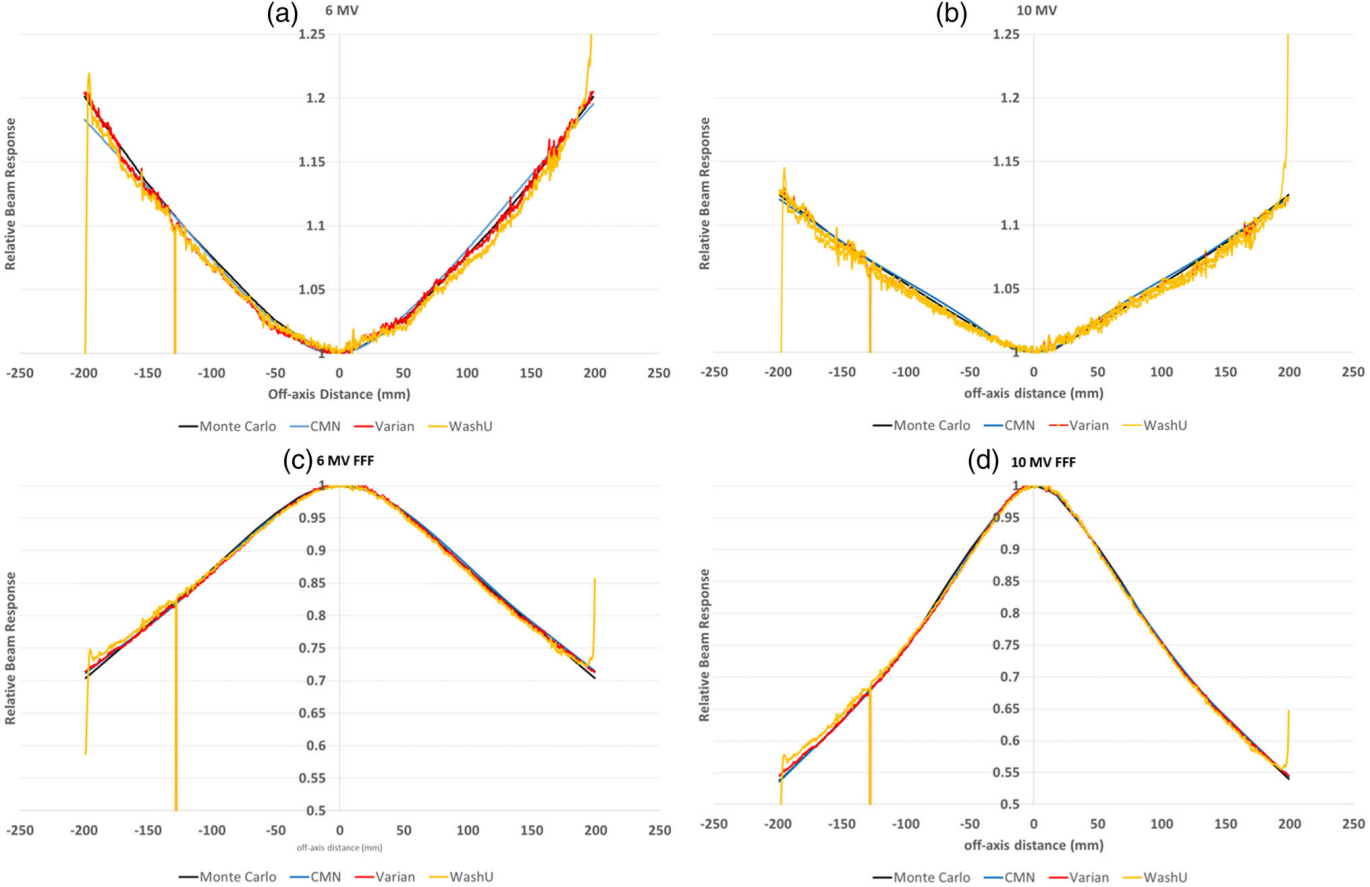
Crossplane central axis profile for the (a) 6 MV, (b) 10 MV, (c) 6 MV FFF, and (d) 10 MV FFF beam‐responses measured with all four methods. *Note*: CMN direct is the directly measured data points, while CMN (fitted) is the fit to these data points to translate the method onto all pixels

Figure 10 shows the 1D crossplane beam‐response profiles generated by all four methods for flattened and FFF beams. Qualitatively, all methods provide similar shaped profiles to each other for all four beams. However, the results begin to diverge outside ± 150 mm, especially for the FFF beams. However, this region is of least importance for QA applications as it is outside a 30 × 30 cm^2^ field size. It is worth reminding from Part 1 of this study^12^ that the Monte Carlo beam‐response is idealized, in that perfect beam symmetry and ideal beam energy is assumed. As this will not actually be the case for a real beam as used with the empirical PSM methods, then any real asymmetries or beam energy variation from ideal will result in variation in the plots of Figure 10. This is a form of inaccuracy in the Monte Carlo method.

Figure 10 shows the same noise in the beam‐response for the WashU and Varian methods as presented in Part 1 of this study.^12^ These new results show that this noise is present for all beams and not just 6 MV. Also present in all energies are the errors associated with dead pixels for the WashU and Varian methods and the errors toward the extremities of the images for the WashU method that were explained in Yaddanapudi et al.^5^

Table 2 shows clinically excellent agreement in measured symmetry between the IC Profiler and the CMN method with all beams and all planes within 0.2%. This is in agreement with the results of Barnes et al.^7^ that were directly measured at 10 cm off‐axis points. The agreement between the Varian and WashU methods and the Profiler is within 0.9% and 2.2%, respectively. This is expected considering the noisy beam‐response presented previously in Part 1 of this study^12^ and here in Figure 10. It is expected that agreement would improve if the beam‐response were smoothed post‐processing. Results were presented here without smoothing to demonstrate the clinical significance of the pixel sensitivities not completely captured in the PSM and hence manifesting as noise in the beam‐response.

**TABLE 2:** Symmetry measured with IC profiler and with the beam‐response calculated from each method of pixel‐sensitivity‐map (PSM) determination for all available photon beams

**Beam**	**Plane**	**Profiler**	**CMN**	**Varian**	**WashU**
6 MV	Inplane	100.4	100.4	101.1	101.7
	Crossplane	100.4	100.6	101.2	101.3
10 MV	Inplane	100.4	100.2	100.9	101.3
	Crossplane	100.2	100.1	101.1	101.3
6 MV FFF	Inplane	100.3	100.2	100.8	102.4
	Crossplane	100.6	100.7	101.1	102.5
10 MV FFF	Inplane	100.8	100.8	101.2	103.0
	Crossplane	101.1	101	101.5	102.7

Monte Carlo results were not presented in Table 2 as they are inherently symmetric. This weakness of the Monte Carlo method has been discussed in detail in Part 1 of this study,^12^ but the results of Table 1 which show measured asymmetry from all methods provide an example why an artificially symmetric beam‐response is problematic for a QA application.

#### 3.2.2 Inter‐method comparison of PSM

Similar to the 6 MV results presented in Part 1 of this study,^12^ when PSMs generated by the empirical methods are compared to the Monte Carlo method for multiple beams, the mean and standard deviation metrics are heavily skewed to the point of being uninformative. This is due to extreme results caused at the extremities of the image where there are edge effects, in the corners where agreement is influenced by the presence of the primary collimator and by dead pixels, which the Varian and WashU methods appear to include in the beam‐response rather than the PSM. For these reasons, the median percent deviation is presented in Table 3.

**TABLE 3:** Inter‐method pixel‐sensitivity‐map (PSM) median percent deviation compared to the Monte Carlo result for the corresponding beam

	%Deviation		
Beam (MV)	CMN	Varian	WashU
6	−0.36	0.24	0.74
6 FFF	−0.43	0.27	−0.4
10	−0.21	0.24	0.55
10 FFF	−0.32	0.19	−0.68

The median percent deviation for the CMN method with the Monte Carlo method as presented in Table 3 is negative for all beams, but relatively consistent in magnitude between −0.21 and −0.43%. This indicates an under representation of the pixel gain by the CMN method and indicates that the findings of Part 1 of this study^12^ for the CMN PSM are consistent with other beams.

The PSM median percent deviation for the Varian method compared to the Monte Carlo method across beams is most consistent of all methods and consistently the smallest magnitude of all methods, ranging between 0.19% and 0.27%. The WashU median agreement is least consistent with FFF results, having a negative value and flattened beams a positive value with magnitude ranging up to 0.74% which is the highest disagreement of all methods.

### 3.3 Dose rate dependency

**FIGURE 11:**
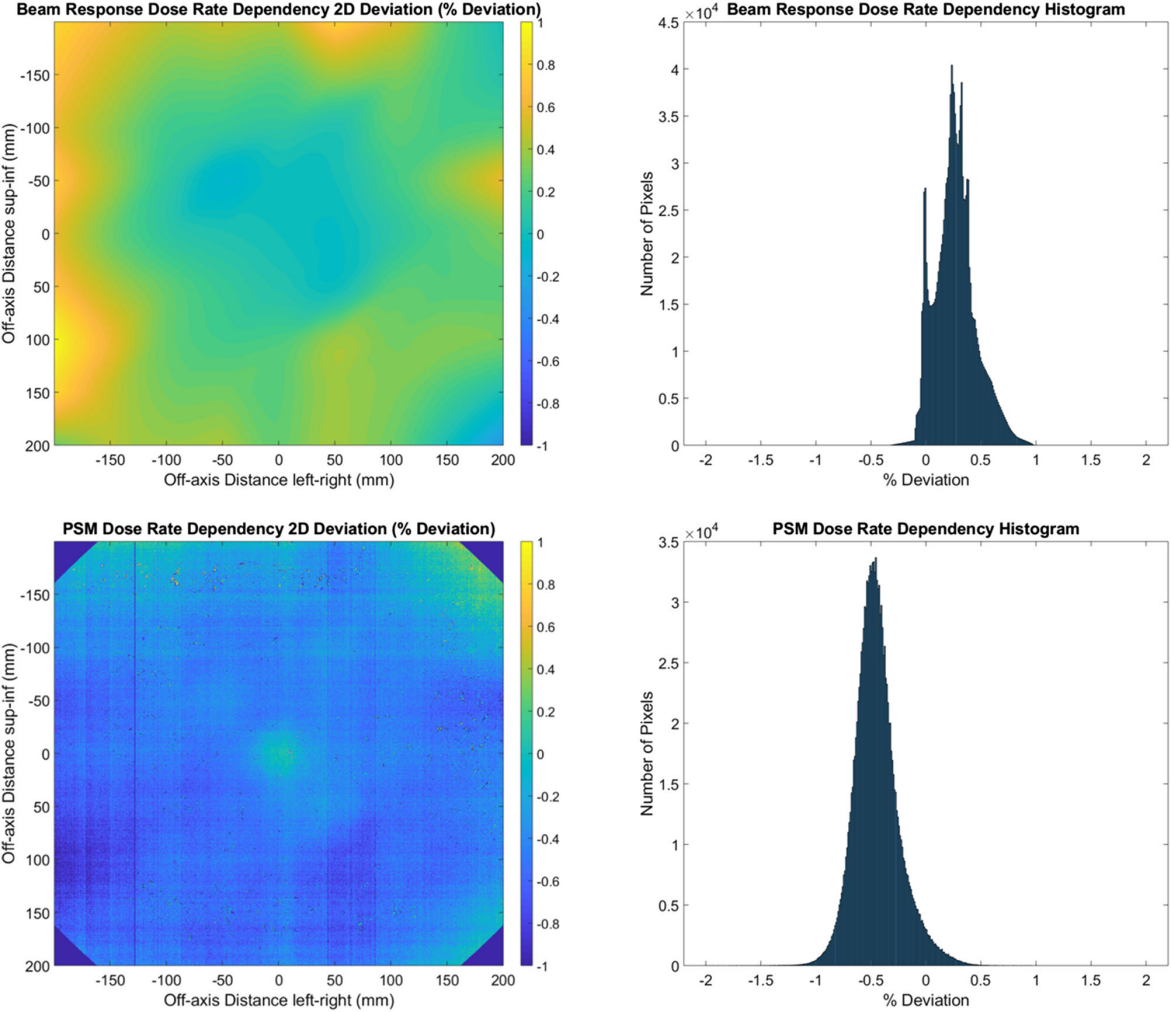
Dose rate dependency measured using the 10 MV FFF beam comparing 2400 MU/min and 400 MU/min via the CMN pixel‐sensitivity‐map (PSM) method. Beam‐response 2D percent deviation (top left), Beam‐Response percent deviation histogram (top right), PSM 2D percent deviation (bottom left), and PSM percent deviation histogram (bottom right)

Figure 11 presents the 2D comparison between the beam response and PSM for the 10 MV FFF beam measured using the CMN method at 400 MU/min and 2400 MU/min dose rates. The results show generally consistent agreement for both PSM and beam response across the image with an approximate 0.5% offset and ± 0.5% spread. The median percent deviation is 0.26% for the beam response and −0.47% for the PSM. The worst agreement is observed toward the periphery of the image, which has least clinical importance.

The CMN method measures the beam response and the PSM is then generated by dividing the raw image by the beam response. Therefore, if there was no dependency on the dose rate for the beam response then the percent deviation histogram would be expected to be centered on zero to within the CMN method repeatability uncertainty presented in the Part 1 study.^12^ The beam response deviations of Figure 11 are marginally outside repeatability (median deviation of 0.26% compared to 95% of pixels repeatable to within 0.21% as per the Part 1 study) hence indicating a small beam‐response dose rate dependence. This could be due to small differences in the beam at the 400 MU/min dose rate that have not been accurately characterized by the 2400 MU/min flood field that was removed from each image as part of the CMN method. The TrueBeam system only allows the flood field to be taken at nominal dose rate of 2400 MU/min for the 10 MV FFF beam.

Since the PSM is generated in the CMN method by removal of the measured beam response then if there was no PSM dependency on dose rate then the PSM results of Figure 11 would be expected to be, within repeatability uncertainty, the inverse of the beam response results. Both the 2D deviation plots and histogram qualitatively demonstrate this to some extent with areas of disagreement in the same parts of the image in the 2D deviation plots. However, the slightly greater median percent deviation magnitude associated with the PSM compared to the beam response indicates a small dependency, likely clinically insignificant, of the PSM with dose rate at least for the CMN method and 10 MV FFF beam.

## 4 DISCUSSION

The results of Figures 2, 3, 4, 5, 6, 7, 8, 9 show that there is a dependence of PSM on the photon beam used for all methods evaluated. The variations observed with beam are outside the repeatability results presented in Part 1 of this study.^12^ This indicates that the dependency on beam is real and not simply due to uncertainties introduced by the method itself. The dose rate dependency experiment suggests that the dose rate may make a small contribution to this dependency. Although the experiment is not considered conclusive these findings are in agreement with those of Xu et al.^14^ who presented a small dose rate dependency for FFF beams for the EPID panel used in this study. Also, reduction of the dose rate reduces the pulse frequency and not the dose‐per‐pulse. A definitive study on dose rate dependency would ideally inform on both pulse frequency and dose‐per‐pulse dependence. Further investigations of the PSM beam dependencies are recommended for further work. The high agreement between PSMs from different beams for the Varian method and also the CMN method at the directly measured points suggests that, if anything the underlying PSM has only small beam dependence, likely insignificant in the clinical context and that the dependence introduced by these two methods is also small. This supports the hypothesis that, at least for the Varian and CMN methods, a PSM could be derived from a single photon beam and be applied to all other photon beams. This furthers the evidence presented by Bin Cai et al.^6^ and Ahmad et al.^11^ that the PSM is beam independent. A single PSM used for multiple beams would reduce the required time to obtain and maintain any potential PSM calibration.

The beam‐response profiles of Figure 10 reaffirm the findings of Part 1 of this study^12^ in that qualitatively, each method of PSM determination provides a similar shape beam‐response, but that the WashU method and Varian method are unexpectedly noisy and the WashU method becomes inaccurate toward the extremities of the imager panel as previously presented in the literature.^5^ The results presented here in Part 2 suggest that these effects are independent of the photon beam used to measure the PSM and hence generate the beam‐response.

In this study, the measured beam symmetry was used as a quantitative method for assessing the accuracy of the beam‐response generated by each method. The results suggested that in this regard the CMN method is superior and highlighted the drawback of the Monte Carlo method, where perfect symmetry is inherent. Significant disagreement in the measured symmetry was observed for the Varian and WashU methods, which will at least partially be due to the noise inherent in the beam‐response from these methods. A simple solution to this problem is to smooth the beam‐responses prior to applying the symmetry metric, however, using this metric with the noise still included, provided a demonstration as to a potential clinical effect of this noise. Due to the mutual dependence of the PSM and beam‐response (an error in one leads to an inaccuracy in the other) the effect of noise in the beam‐response will be inaccuracy in the corresponding PSM.

While symmetry was chosen in this study as a measure of clinical significance, the utility of each method will likely be application specific. As such, other methods of assessment should be considered specific to the application.

In general, the PSM generated by the Varian method agrees best with the Monte Carlo method in terms of median agreement, followed by the CMN method and the WashU method. For all methods, the median percent deviation is always within 0.5%, except three out of the four beams with the WashU method in which case these beams are within 0.75%.

With multiple beams and multiple PSM methods, there are many relationships within the results that have not been investigated in this study and these are now recommended for further work. One example is investigation of the effect on measured symmetry of smoothing the Varian and WashU beam‐responses. Another is investigating whether methods can be modified to assign dead pixels and the primary collimator correctly to the PSM and beam‐response, respectively. The Monte Carlo method could also be improved with a greater number of histories, but its flaws of assuming ideal beam symmetry and beam energy are inherent. The CMN method could potentially be investigated to see whether changing the number of directly measured data points improved the quality of the fit, particularly in the extrapolated regions at the extremity of the image or conversely whether a reduced number of directly measured points could reduce the required measurement time without affecting accuracy. The effect of dark field calibration has not been assessed in this study, although the WashU and Varian methods utilize the ABDF technique that takes dark field images during the data acquisition process so in theory should be unaffected. Lastly, other clinical significance metrics rather than beam symmetry could be investigated to inform on the performance of each PSM method. A simple way of doing this would be to try each PSM method for any PSM application developed and benchmark the results of the application against an independent system. Since the work was only performed on a single EPID panel then a follow‐up study to verify these studies results on multiple EPID panels is also suggested.

Based upon the findings from both parts 1 and 2 of this study, the choice of which PSM method is best for clinical use is likely application specific. The required accuracy should be weighed against the time required to acquire the PSM and consideration should also be given to the EPID panel‐type used, likely field sizes to be measured and to whether multiple photon beams will be utilized.

## 5 CONCLUSIONS

Strengths and weaknesses of each of the PSM methods available to this study have been expanded upon from Part 1 of this study and the beam dependence of the PSM and of the four available methods for determining it has been investigated. Non‐conclusive evidence is presented that, at least for photon beams, that the actual PSM may be independent of photon beam.

## CONFLICT OF INTEREST

The authors declare that there is no conflict of interest that could be perceived as prejudicing the impartiality of the research reported.

## AUTHOR CONTRIBUTIONS

Michael Barnes: Had the original idea for the study, performed data acquisition, and analysis and primarily wrote the manuscript.

Baozhou Sun: Provided the WashU and Varian PSM methods and for these methods performed both post processing of data to generate PSM and wrote the relevant methods section of the manuscript. Baozhou also provided scientific input into the study and writing of the manuscript.

Brad Oborn: Provided the Monte Carlo simulations, wrote the Monte Carlo related methods sections and provided scientific input into the study and writing of the manuscript.

Bishnu Lamichhane: Performed the 2D curve fitting required for the CMN method and wrote the corresponding section in the methods as well as providing scientific input into the study and writing of the manuscript.

Matthew Schmidt, Stuart Szwec, Bin Cai, Fred Menk and Peter Greer: All provided scientific input into the study and writing of the manuscript.

## Supporting information

Supporting Information
