# Supplementary material for: Determination of the electronic portal imaging device pixel‐sensitivity‐map for quality assurance applications. Part 2: Photon beam dependence
Source: J Appl Clin Med Phys. 2022 Apr 15;23(6):e13602. doi: 10.1002/acm2.13602 (PMC9195019; doi:10.1002/acm2.13602)
Supplement: Supplementary file 1 — Supporting Information [file ACM2-23-e13602-s001.docx]

# Supplementary material

**Beam dependent inter method PSM comparison with the Monte Carlo method**

**6 MV FFF**


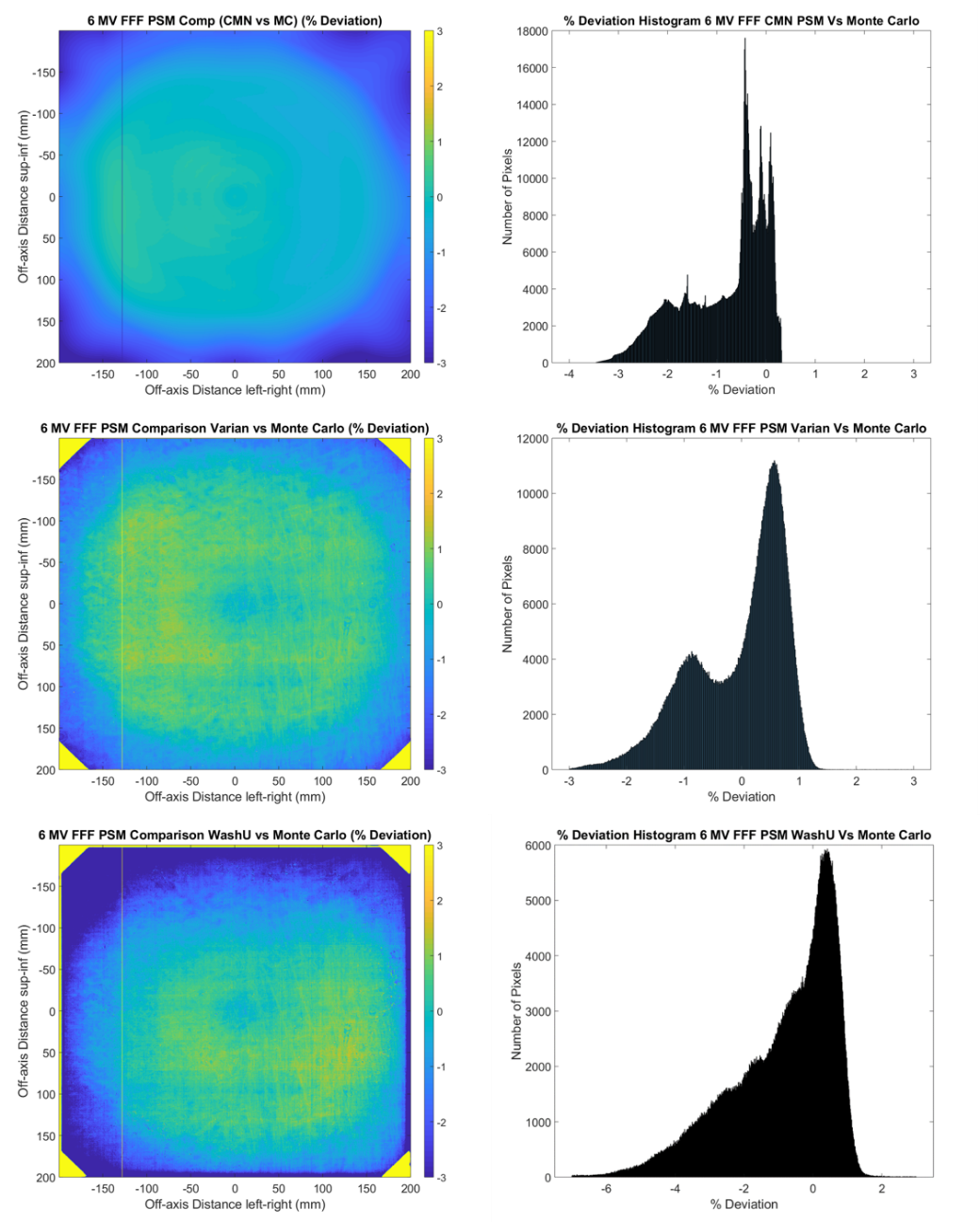


6 MV FFF 2D % deviation in PSM between each method compared to Monte Carlo (CMN top, Varian middle and WashU bottom). % Deviation maps (left) and corresponding histograms (right). Note: x-axis scale on WashU histogram is different to the other methods.


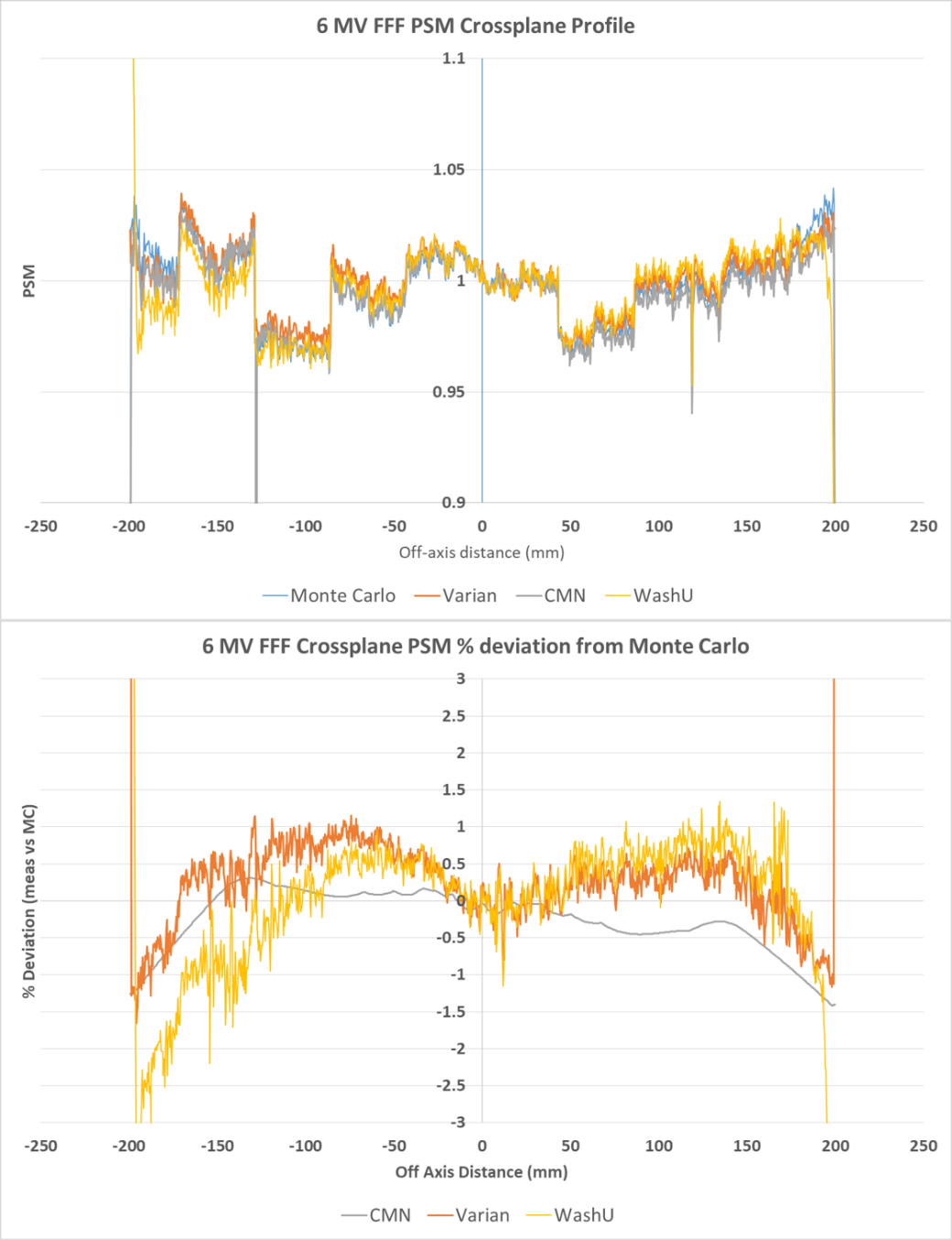


6 MV FFF 1D central axis crossplane PSM profile comparison between methods. Crossplane Profiles (top) and % Deviation compared to Monte Carlo (bottom)

**10 MV**


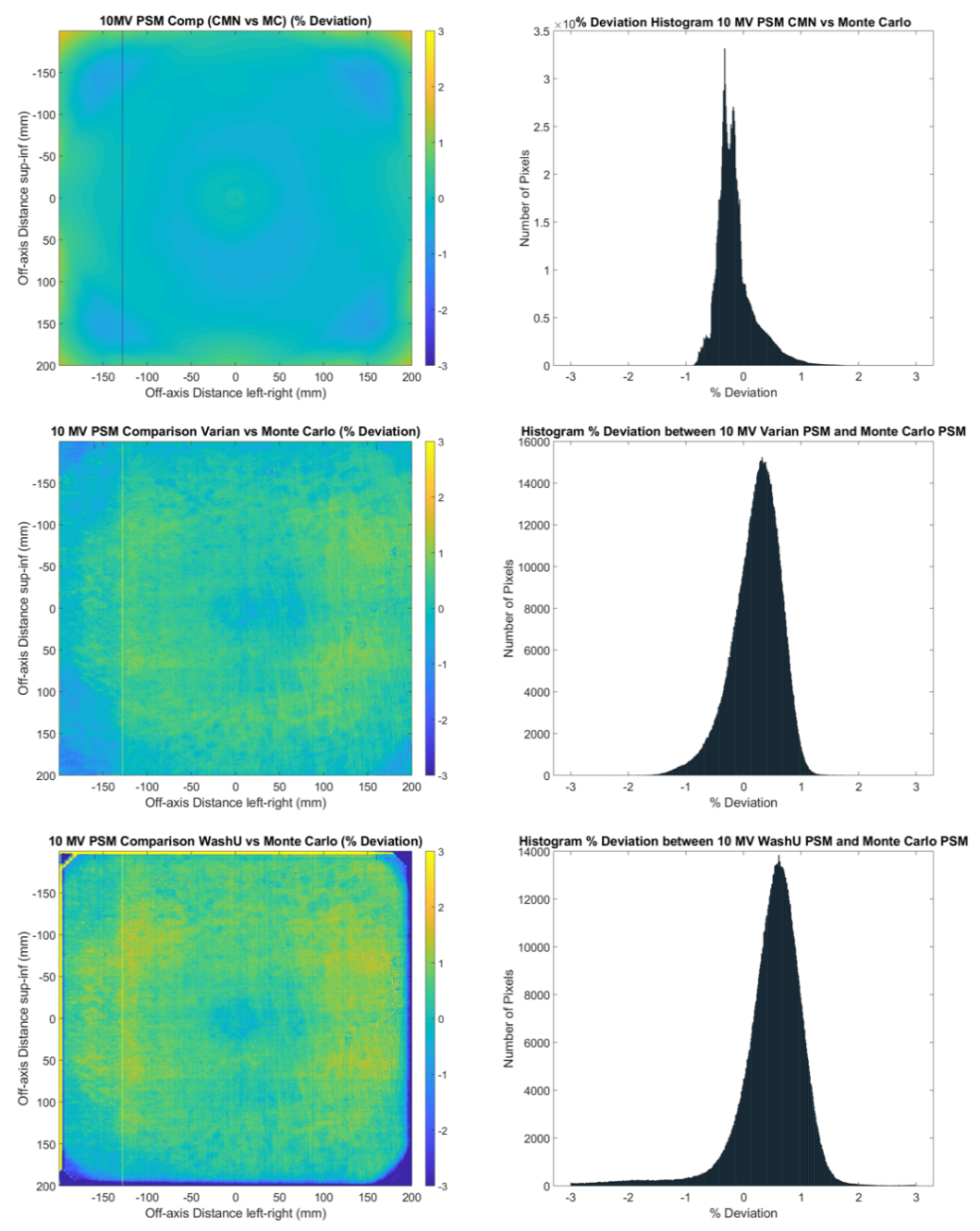


10 MV 2D % deviation in PSM between each method compared to Monte Carlo (CMN top, Varian middle and WashU bottom). % Deviation maps (left) and corresponding histograms (right)


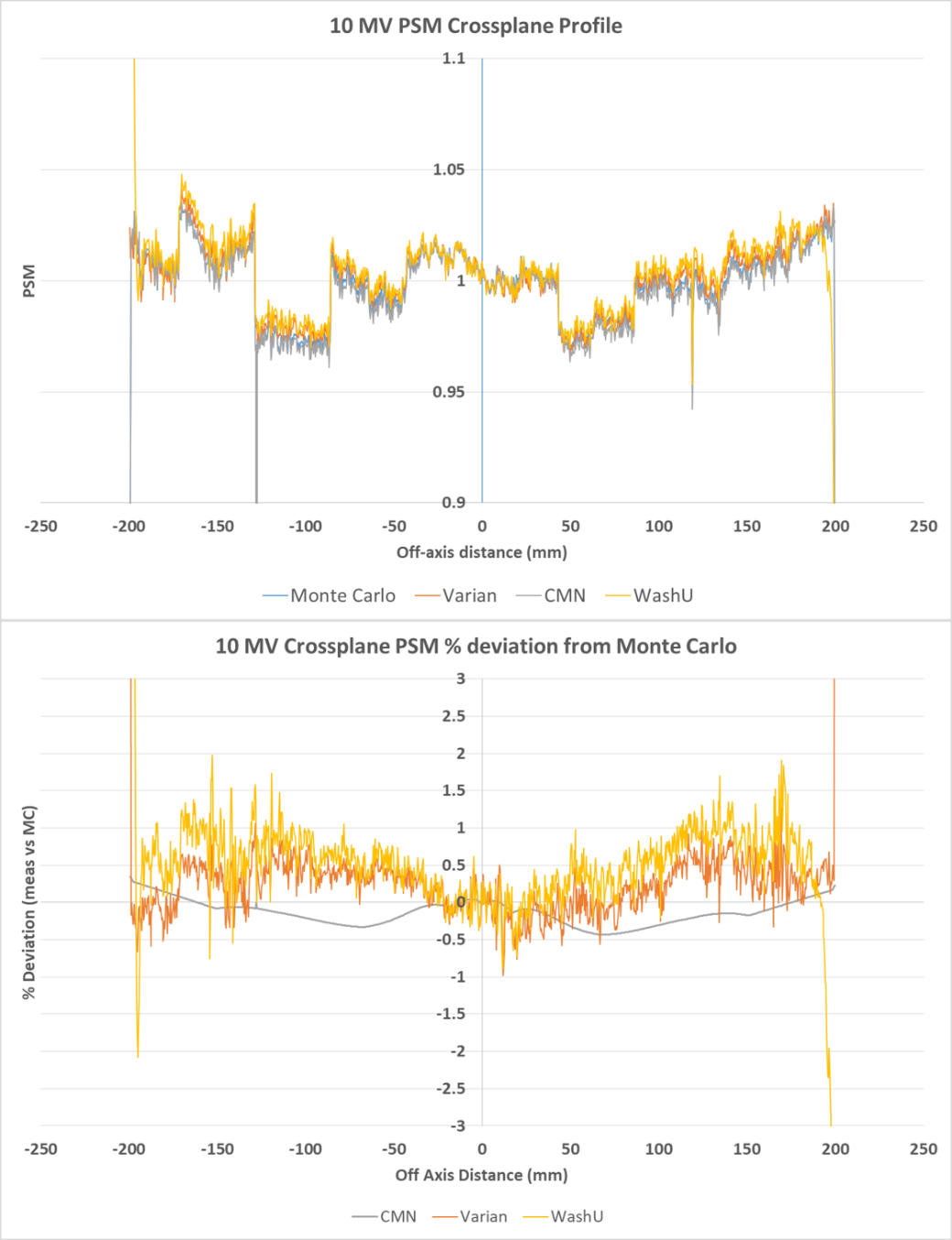


10 MV 1D central axis crossplane PSM profile comparison between methods. Crossplane Profiles (top) and % Deviation compared to Monte Carlo (bottom)

**10 MV FFF**


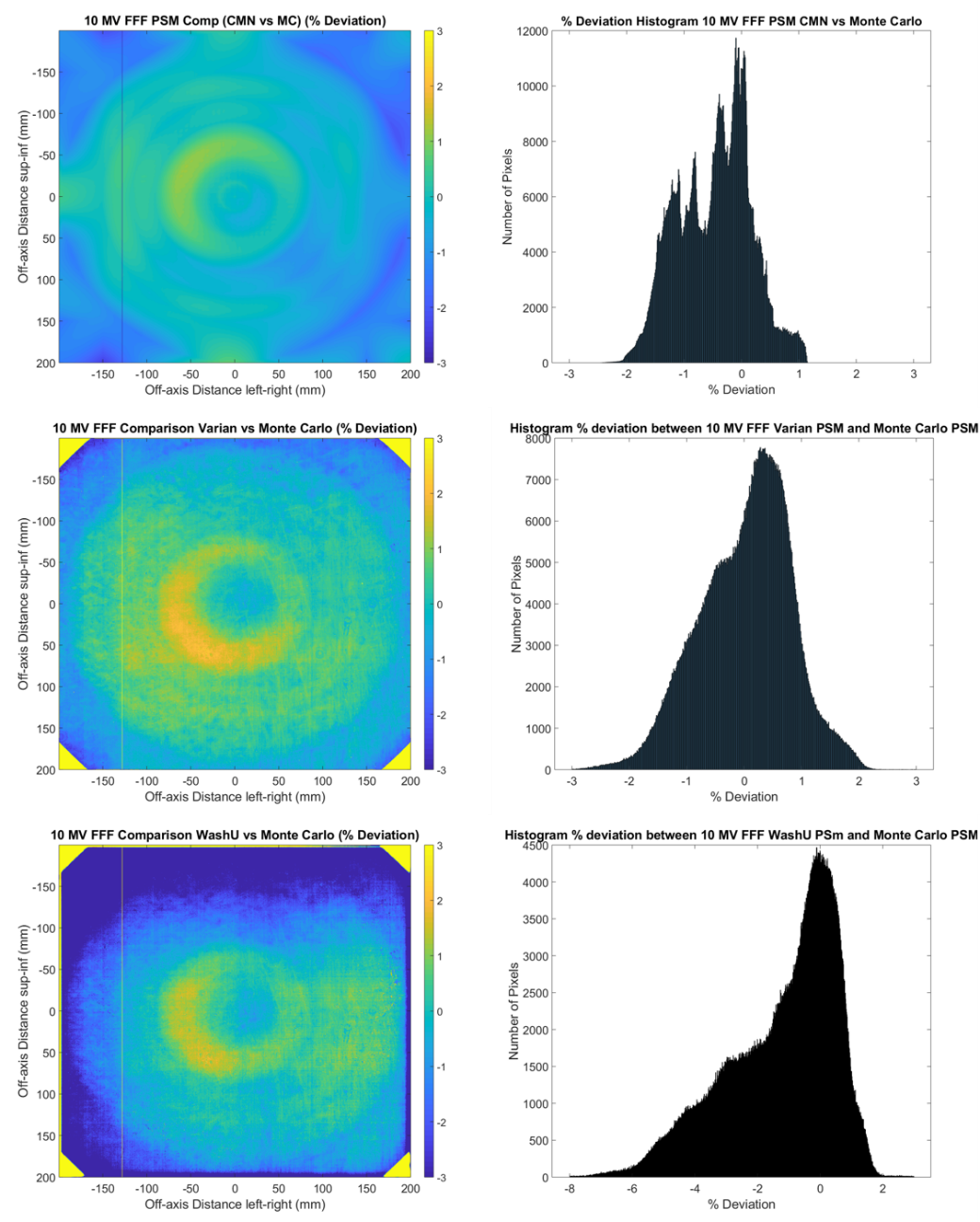


10 MV FFF 2D % deviation in PSM between each method compared to Monte Carlo (CMN top, Varian middle and WashU bottom). % Deviation maps (left) and corresponding histograms (right). Note: x-axis scale on WashU histogram is different to the other methods**.**


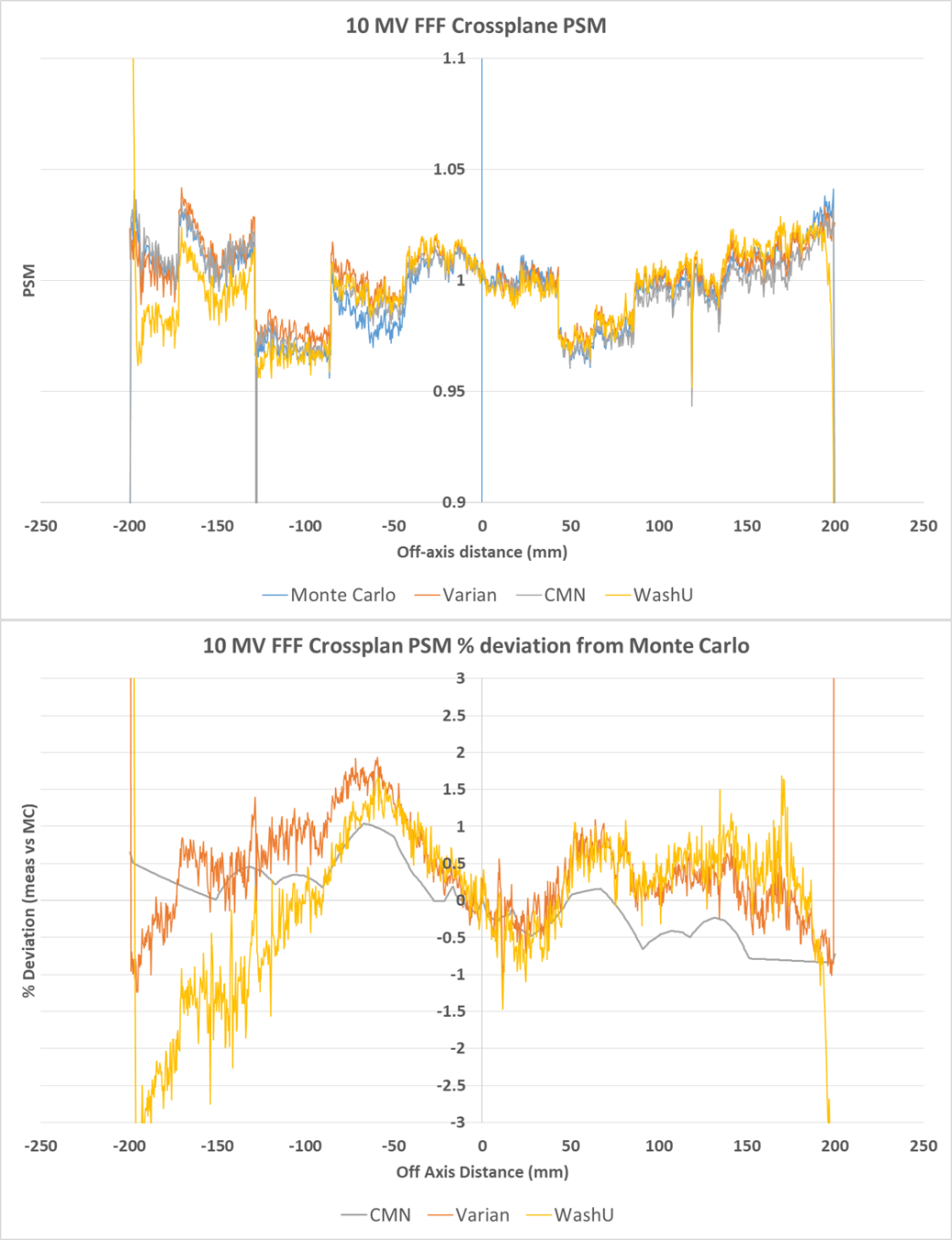


10 MV FFF 1D central axis crossplane PSM profile comparison between methods. Crossplane Profiles (top) and % Deviation compared to Monte Carlo (bottom)
